# Platelet-driven coagulopathy in COVID-19 patients: in comparison to seasonal influenza cases

**DOI:** 10.1186/s40164-021-00228-z

**Published:** 2021-05-31

**Authors:** Jianguo Zhang, Xing Huang, Daoyin Ding, Zhimin Tao

**Affiliations:** 1 Jiangsu Province Key Laboratory of Medical Science and Laboratory Medicine, School of Medicine, Jiangsu University, Zhenjiang, 212013 Jiangsu China; 2 Department of Critical Care Medicine, The Affiliated Hospital, Jiangsu University, Zhenjiang, 212001 Jiangsu China; 3 Center for Evidence-Based and Translational Medicine, Zhongnan Hospital of Wuhan University, Wuhan, 430071 China; 4 Department of Critical Care Medicine, The First People’s Hospital of Jiangxia District, Wuhan, 430200 Hubei China

**Keywords:** COVID-19, SARS-CoV-2, Intensive care unit, Coagulation disorder, Influenza

## Abstract

**Background:**

One year into the coronavirus diseases 2019 (COVID-19) pandemic we analyzed the blood coagulopathy in severe and non-severe COVID-19 patients and linked to those of influenza patients for a comparative study.

**Methods:**

We reported 461 COVID-19 patients and 409 seasonal influenza patients admitted at separated medical centers. With their demographic data and medical history, hematological profiles with coagulation characters were emphasized, and compared between two cohorts before and after treatment.

**Results:**

For 870 patients included in this study, their median age was (64.0, 51.0–76.0), and among them 511 (58.7%) were male. Hypertension, diabetes, cardiovascular diseases, and bronchitis constituted the leading comorbidities. Upon hospital admission blood test results differentiated COVID-19 patients from influenza cases, and for COVID-19 patients, leukocytosis, neutrophilia, lymphocytopenia, and thrombocytopenia were associated with disease severity and mortality. In addition, COVID-19 cohort demonstrated a prolonged prothrombin time (PT) and activated partial thromboplastin time (aPTT), increased INR, shortened thrombin time and decreased fibrinogen, compared to those in influenza cohort, leaving D-dimer levels indistinguishably high between both cohorts. Platelet hyperreactivity in COVID-19 is more evident, associated with worse hyper-inflammatory response and more refractory coagulopathy. For severe COVID-19 patients administered with anticoagulants, bleeding incidence was substantially higher than others with no anticoagulant medications.

**Conclusions:**

Comparison of coagulation characteristics between COVID-19 and influenza infections provides an insightful view on SARS-CoV-2 pathogenesis and its coagulopathic mechanism, proposing for therapeutic improvement.

**Supplementary Information:**

The online version contains supplementary material available at 10.1186/s40164-021-00228-z.

## Background

One year after coronavirus disease 2019 (COVID-19) surfaced, the worldwide infection toll has reached over 100 million people as the public health managements face an unprecedented challenge amid this global pandemic[1]. The disease is caused by severe acute respiratory syndrome coronavirus 2 (SARS-CoV-2), which genetically falls into the β-genus of coronavirus family [2, 3]. Appearing a particle of ~ 60–140 nm with 9–12 nm spikes protruding from its surface, SARS-CoV-2 showed 96.2% genomic identity to RaTG13 detected in *Rhinolophus affinis *[3, 4].

SARS-CoV-2 relies on angiotensin-converting enzyme 2 (ACE2) in the host for cellular entry, and this fact mirrors the susceptibility of various organs to this viral infection [4–6]. RNAs of SARS-CoV-2 have been detected in a variety of clinical specimens, including nasal and pharyngeal swabs, bronchoalveolar lavage fluid, sputum, blood and feces [7]. Pieced together, those findings illustrate the invasive pathway of SARS-CoV-2 into human through inhalation to cause primary lung damage, followed by secondary injuries to other organs (especially heart, liver, kidney), via either direct virus attack through blood flow or indirect insults due to systemic inflammatory response.

Studies during early COVID-19 breakout noticed heightened D-dimer levels in COVID-19 patients, especially those under critically ill conditions [8–10]. Coagulation characters in COVID-19 patients have been examined since then, where increased levels of D-dimer, fibrin degradation product, prolonged prothrombin time (PT) and activated partial thromboplastin time (aPTT) were associated with the worsened condition in COVID-19 patients [11]. Anticoagulation medication using low molecular weight heparin (LMWH) lowered the 28-day mortality in COVID-19 patients with strikingly elevated D-dimer levels [12]. Concurrently, autopsy studies on deceased COVID-19 patients revealed that thrombosis was frequently observed in arteries and veins of different organs, predominantly lung and heart where viral RNA may or may not be detected [13–16]. Thus pulmonary coagulopathy contributed to the COVID-19 death, despite the usage of therapeutic anticoagulation [14, 16].

Coagulation disorders are associated with a diversity of viral or non-viral diseases, which depend on many factors such as the specific pathogenesis and the underlying condition of individual patient [17]. Coagulopathic characters, such as increased D-dimer concentration and von Willebrand factor activity, are also common complications in influenza infection, triggering life-threatening cytokine storm and vascular thrombosis [18]. Previously, we reported the resemblance and difference between clinical features of COVID-19 and influenza, showing that both viral infections lead to blood abnormality, including leukocytosis, neutrophilia and lymphocytopenia, although their frequencies in both illnesses could be statistically different [19]. In this study we emphasized the coagulation profiles between COVID-19 and influenza patients, with an aim to understand the hematological impact of SARS-CoV-2 infection and the potential therapeutic targets.

## Methods

### Study participants

This study was approved by the First People’s Hospital of Jiangxia District (FPHJD) at Wuhan and the Huangshi City Hospital (HCH) of Hubei Province, and by the Affiliated Hospital of Jiangsu University (AHJU) in Zhenjiang City of Jiangsu Province, in China, respectively. COVID-19 cases were hospitalized at FPHJD and HCH during January 2020 to April 2020, and influenza patients were received at AHJU during January 2018 to April 2020. For all patients, exclusion criteria were as follows: (1) patients with malignant tumors or immunodeficiency; (2) patients with genetic or acquired coagulation disorders; (3) patients with hepatic dysfunction; (4) patients on medications that may affect the blood clotting. As a result, 461 COVID-19 patients were selected, including 209 patients in the ICU and 252 patients in the non-ICU isolation ward. In parallel, 409 influenza patients were collected. For both cohorts, patient information remains anonymous, and written consent was waived by Ethics Commission of FPHJD, HCH and AHJU, respectively.

### Patient procedure

COVID-19 patients were received at FPHJD and HCH, diagnosed by following a standard procedure [20]. At the same time, all COVID-19 patients upon hospital admission were tested for influenza infection using IgM antibody detection against Flu A/B through indirect immunofluorescence assay (Nine respiratory pathogens IgM antibody detection kit, Zhengzhou Autobio Diagnostics Co., China). Patients included in the COVID-19 cohort of this study were all tested influenza virus A/B negative. The confirmed patients were treated with antiviral drugs, including oseltamivir, arbidol, and ribavirin. For the severe patients who were admitted into the Intensive Care Unit (ICU) by following the published criteria [20], where they were receiving antibiotic treatment (sulperazone, linezolid), antifungal therapy (fluconazole, caspofungin), corticosteroid therapy, and respiration-assisted ventilation. For each ICU patient, low molecule weight heparin (LMWH, 4–6 kDa) was administered 5000 IU per day via subcutaneous injection (heparin sodium for injection, manufactured by Jilin Huakang Pharmaceutical Co. Ltd., China), unless a bleeding risk was assessed. In parallel, influenza patients were diagnosed using detection kit of serum IgM antibodies against respiratory viruses based on an immunofluorescence assay (EUROIMMUN, Germany). 389 patients (95.1%) were infected with influenza A virus (H1N1 and H3N2), 327 (80.0%) with influenza B, and among them 307 (75.1%) were co-infected. Influenza patients were hospitalized at AHJU, where oxygen therapy was applied along with ribavirin or oseltamivir antiviral treatment, and none of them was transferred to ICU. For all patients, blood cell analysis was conducted by automated hematology analyzer (SYSMEX 800i, Japan; Mindray BC-5300, China), and the biochemical indicator was also analyzed (Toshiba TAB2000, Japan; Beckman AU5800, USA). Hemostatic drugs, tranexamic acid (1 g via intravenous infusion) or/and hemocoagulase (1 unit via intramuscular injection), were administered for patients who bled, and component blood transfusion of plasma, cryoprecipitate, or platelet was applied once necessary.

### Date collection and analysis

Demographic information, medical history and blood testing results of COVID-19 and influenza patients were obtained and compared. All blood parameters were collected from patients upon hospital admission, and for blood testing after treatment, we adopted the last data of patients before their discharge from the hospital. For deceased patients, data after treatment were not collected. The categorical variables were described as frequency rates and percentages, and continuous variables were applied to describe the median and quartile range (IQR) values. Comparison of continuous variables between two groups was analyzed with Mann–Whitney test. Repeated measurements (non-normal distribution) were used following a generalized linear mixed model. χ^2^ test was used to compare the proportion of categorical variables, and the Fisher exact test was employed when data was limited. All statistical analyses were performed using GraphPad. A two-sided a of less than 0.05 was considered statistically significant.

## Results

### Comparison of baseline characteristics between COVID-19 and influenza patients upon admission

In this multicenter study, a total of 870 patients were included, of which 461 were diagnosed COVID-19 and 409 diagnosed seasonal influenza (with influenza virus A/B) upon hospital admission. Compared to the influenza cohort, the COVID-19 cohort showed a significantly shorter time interval from disease onset to hospital admission, lower median age, and the male gender ratio, but much higher mortality (Table 1). Within COVID-19 cohort, there were 252 mild patients (non-ICU group) who stayed in isolation ward until hospital discharge, and 209 severe patients later transferred to ICU for treatment (ICU group) where 90 patients unfortunately died. No statistical difference in time interval from disease onset to hospitalization was found between different groups within COVID-19. The median age of patients jumped from non-ICU, ICU survivor to ICU non-survivor, while the ratio of male gender was significantly higher in ICU group than in non-ICU group but remained indistinguishable between ICU survivors and non-survivors (Table 2). For both cohorts, hypertension, diabetes, cardiovascular diseases and bronchitis took up the primary comorbidities, echoing with our previous findings [19]. COVID-19 patients with co-existing hypertension experienced an unfavorable prognosis for disease severity and mortality, while in contrast chronic bronchitis did not contribute to disease development of COVID-19 patients (Table 2). In addition, diabetes and cardiovascular were risk factors for COVID-19 severity but not mortality.

**Table 1:** Baseline characteristics, blood parameters and coagulation factors in the COVID-19 cohort, compared to those in the influenza cohort

	Normal	Total (n = 870)	COVID-19 (n = 461)	Influenza (n = 409)	*p* value
Onset to hospitalization, day		4.0 (3.0–5.0)	4.0 (3.0–5.0)	5.0 (4.0–6.0)	< 0.0001
Age		64.0 (51.0–76.0)	58.0 (47.0–70.0)	69.0 (57.0–79.0)	< 0.0001
Gender, male N (%)		511 (58.7%)	250 (54.2%)	261 (63.8%)	0.004
Mortality, N (%)		107 (12.3%)	98 (21.3%)	9 (2.2%)	< 0.0001
*Comorbidity*					
Hypertension		281 (32.3%)	120 (26.0%)	161 (39.4%)	< 0.0001
Diabetes		97 (11.1%)	71 (15.4%)	26 (6.4%)	< 0.0001
Cardiovascular diseases		96 (11.0%)	53 (11.5%)	43 (10.5%)	0.644
Bronchitis		76 (8.7%)	34 (7.4%)	42 (10.3%)	0.131
*Blood parameters*					
White blood cells, × 10^9^/L	3.5–9.5	6.8 (5.0–9.2)	6.4 (4.9–8.7)	7.3 (5.3–9.9)	< 0.001
> 9.5		206 (23.7%)	98 (21.3%)	108 (26.4%)	0.075
Neutrophils, × 10^9^/L	1.8–6.3	5.0 (3.4–7.4)	4.7 (3.1–6.9)	5.5 (3.6–7.8)	0.003
> 6.3		298 (34.3%)	143 (31.0%)	155 (37.9%)	0.033
Lymphocytes, × 10^9^/L	1.1–3.2	1.0 (0.7–1.5)	1.0 (0.6–1.4)	1.1 (0.7–1.5)	0.001
< 1.1		458 (52.6%)	270 (58.6%)	188 (46.0%)	< 0.001
Monocytes, × 10^9^/L	0.1–0.6	0.5 (0.3–0.7)	0.5 (0.3–0.6)	0.5 (0.4–0.8)	< 0.0001
> 0.6		263 (30.2%)	121 (26.2%)	142 (34.7%)	0.007
Red blood cells, × 10^12^/L	4.3–5.8	4.1 (3.5–4.5)	4.0 (3.4–4.5)	4.2 (3.7–4.6)	< 0.001
< 4.3		546 (62.8%)	300 (65.1%)	246 (60.1%)	0.133
Hemoglobin, g/L	130–175	123.0 (106.0–136.0)	120.0 (100.0–135.0)	126.0 (111.5–137.0)	< 0.001
< 130		541 (62.2%)	299 (64.9%)	242 (59.2%)	0.084
Hematocrit, %	40–50	36.9 (32.4–40.7)	36.0 (31.6–40.0)	37.8 (33.9–41.6)	< 0.0001
< 40		611 (70.2%)	346 (75.1%)	265 (64.8%)	0.001
Platelet, × 10^9^/L	125–350	195.0 (145.0–261.3)	191.0 (138.0–260.5)	201.0 (150.5–267.0)	0.064
< 125		146 (16.8%)	94 (20.4%)	52 (12.7%)	0.003
Mean platelet volume, fL	7.4–12.5	10.7 (9.9–11.6)	10.7 (10.0–11.5)	10.7 (9.8–11.6)	0.618
> 12.5		85 (9.8%)	47 (10.2%)	38 (9.3%)	0.654
*Coagulation factors*					
Prothrombin time, s	9–13	12.9 (11.8–14.2)	13.3 (12.3–14.2)	12.2 (11.4–14.1)	< 0.0001
> 13		418 (48.0%)	268 (58.1%)	150 (36.7%)	< 0.0001
INR	0.8–1.2	1.1 (1.0–1.2)	1.1 (1.0–1.2)	1.0 (1.0–1.2)	0.007
> 1.2		179 (20.5%)	88 (19.1%)	91 (22.2%)	0.250
aPTT, s	23.3–32.5	29.2 (26.4–32.1)	30.4 (28.2–32.7)	27.4 (24.8–31.5)	< 0.0001
> 32.5		196 (22.5%)	119 (25.8%)	77 (18.8%)	0.014
Thrombin time, s	14–21	17.1 (15.9–18.4)	16.4 (15.4–17.6)	17.7 (16.7–19.5)	< 0.0001
> 21		109 (12.5%)	19 (4.1%)	90 (22.0%)	< 0.0001
Fibrinogen, g/L	2–4	4.0 (3.0–5.1)	3.9 (2.8–4.8)	4.2 (3.1–5.3)	< 0.001
> 4		429 (49.3%)	206 (44.7%)	223 (54.5%)	0.004
D-dimer, mg/L	< 0.55	1.10 (0.44–2.80)	1.08 (0.35–3.27)	1.13 (0.49–2.60)	0.555
> 0.55		607 (69.8%)	313 (67.9%)	294 (71.9%)	0.201

**Table 2:** Baseline characteristics, blood parameters and coagulation factors are compared between the non-ICU and the ICU groups within COVID-19 cohort, and between the survivor and the non-survivor groups of COVID-19 ICU patients, respectively

COVID-19 (n = 461)	Normal range	Non-ICU (n = 252)	ICU (n = 209)^*p1*^	*p1* value	ICU (n = 209)		
					Survivor (n = 119)	Non-survivor (n = 90)^*p2*^	*p2* value
Onset to hospitalization, day		4.0 (3.0–5.0)	4.0 (2.0–5.0)	0.254	4.0 (3.0–5.0)	3.5 (2.0–5.0)	0.572
Age		51.5 (39.0–63.0)	67.0 (57.0–76.5)	< 0.0001	63.0 (55.0–76.0)	69.0 (58.8–78.3)	0.043
Gender, male N (%)		122 (48.4%)	128 (61.2%)	0.006	76 (63.9%)	52 (57.8%)	0.371
Mortality, N (%)		8 (3.2%)	90 (43.1%)	< 0.0001	0 (0)	90 (100%)	
*Comorbidity*							
Hypertension		47 (18.7%)	73 (34.9%)	< 0.0001	32 (26.9%)	41 (45.6%)	0.005
Diabetes		27 (10.7%)	44 (21.1%)	0.002	25 (21.0%)	19 (21.1%)	0.986
Cardiovascular diseases		8 (3.2%)	45 (21.5%)	< 0.0001	25 (21.0%)	20 (22.2%)	0.833
Bronchitis		17 (6.7%)	17 (8.1%)	0.570	6 (5.0%)	11 (12.2%)	0.060
*Blood parameters*							
White blood cells, × 10^9^/L	3.5–9.5	6.1 (4.7–7.5)	7.3 (5.0–11.9)	< 0.0001	7.0 (4.9–10.1)	8.6 (5.5–13.0)	0.015
> 9.5		24 (9.5%)	74 (35.4%)	< 0.0001	32 (26.9%)	42 (46.7%)	0.003
Neutrophils, × 10^9^/L	1.8–6.3	4.3 (2.9–5.9)	6.0 (3.6–10.0)	< 0.0001	5.1 (3.4–7.8)	7.3 (4.2–13.0)	0.001
> 6.3		44 (17.5%)	99 (47.4%)	< 0.0001	46 (38.7%)	53 (58.9%)	0.004
Lymphocytes, × 10^9^/L	1.1–3.2	1.1 (0.8–1.6)	0.8 (0.5–1.1)	< 0.0001	0.8 (0.5–1.3)	0.7 (0.4–1.0)	0.010
< 1.1		118 (46.8%)	152 (72.7%)	< 0.0001	78 (65.5%)	74 (82.2%)	0.007
Monocytes, × 10^9^/L	0.1–0.6	0.5 (0.3–0.6)	0.4 (0.3–0.6)	0.464	0.4 (0.3–0.6)	0.4 (0.2–0.7)	0.356
> 0.6		61 (24.2%)	60 (28.7%)	0.274	34 (28.6%)	26 (28.9%)	0.960
Red blood cells, × 10^12^/L	4.3–5.8	4.3 (3.9–4.6)	3.5 (2.9–4.1)	< 0.0001	3.5 (2.9–4.1)	3.4 (2.9–4.2)	0.547
< 4.3		128 (50.8%)	172 (82.3%)	< 0.0001	102 (85.7%)	70 (77.8%)	0.137
Hemoglobin, g/L	130–175	128.0 (115.3–140.0)	107.0 (86.0–124.5)	< 0.0001	108.0 (88.0–125.0)	106.0 (83.8–124.5)	0.941
< 130		134 (53.2%)	165 (78.9%)	< 0.0001	94 (79.0%)	71 (78.9%)	0.986
Hematocrit, %	40–50	37.8 (34.6–40.9)	32.2 (26.7–37.2)	< 0.0001	32.5 (26.5–37.2)	31.9 (26.8–37.1)	0.915
< 40		168 (66.7%)	178 (85.2%)	< 0.0001	103 (86.6%)	75 (83.3%)	0.517
Platelet, × 10^9^/L	125–350	201.0 (152.0–260.0)	173.0 (106.5–269.5)	0.002	197.0 (140.0–276.0)	137.5 (84.8–211.3)	0.001
< 125		27 (10.7%)	67 (32.1%)	< 0.0001	25 (21.0%)	42 (46.7%)	< 0.0001
Mean platelet volume, fL	7.4–12.5	10.5 (9.7–11.2)	10.9 (10.3–12.1)	< 0.0001	10.7 (10.2–11.5)	11.4 (10.4–12.7)	0.004
> 12.5		7 (2.8%)	40 (19.1%)	< 0.0001	16 (13.4%)	24 (26.7%)	0.016
*Coagulation factors*							
Prothrombin time, s	9–13	13.4 (12.6–14.0)	13.2 (12.0–15.0)	0.712	12.7 (11.7–13.9)	14.2 (12.5–16.6)	< 0.0001
> 13		157 (62.3%)	111 (53.1%)	0.046	50 (42.0%)	61 (67.8%)	< 0.001
INR	0.8–1.2	1.1 (1.0–1.1)	1.1 (1.0–1.3)	0.003	1.1 (1.0–1.2)	1.1 (1.0–1.5)	0.005
> 1.2		25 (9.9%)	63 (30.1%)	< 0.0001	27 (22.7%)	36 (40.0%)	0.007
aPTT, s	23.3–32.5	30.1 (28.1–31.5)	31.4 (28.1–36.2)	0.0001	30.7 (28.0–35.3)	31.7 (28.6–37.7)	0.224
> 32.5		31 (12.3%)	88 (42.1%)	< 0.0001	48 (40.3%)	40 (44.4%)	0.551
Thrombin time, s	14–21	15.9 (15.0–17.0)	17.3 (16.1–18.5)	< 0.0001	17.1 (16.3–18.2)	17.5 (16.0–19.0)	0.362
> 21		0 (0)	19 (9.1%)	< 0.0001	8 (6.7%)	11 (12.2%)	0.171
Fibrinogen, g/L	2–4	3.5 (2.6–4.3)	4.2 (3.4–5.5)	< 0.0001	4.2 (3.4–5.4)	4.3 (3.3–5.5)	0.831
> 4		85 (33.7%)	122 (58.4%)	< 0.0001	70 (58.8%)	51 (56.7%)	0.755
D-dimer, mg/L	< 0.55	0.6 (0.2–1.2)	3.2 (1.0–6.2)	< 0.0001	2.2 (0.8–5.2)	3.7 (1.4–7.3)	0.010
> 0.55		134 (53.2%)	179 (85.6%)	< 0.0001	99 (83.2%)	80 (88.9%)	0.245

In this study no ICU patients were admitted in the influenza cohort. Compared to influenza patients, COVID-19 patients in the non-ICU group owned much lowered age and male predisposition but similar mortality, whereas COVID-19 ICU patients had indistinguishable median age and male ratio but much elevated mortality (Additional file 1: Table S1). For both infections, cough, fever, and expectoration constituted major clinical symptoms (results not shown), where the frequency of patients undergoing fever in both cohorts was similar, but the frequency of influenza patients experiencing cough or having phlegm was higher than that of COVID-19 patients. This became in consistency with our previous report [19]. From the onset of disease when typical symptom appeared to the patient hospitalization, the time intervals in influenza cohort were prolonged compared to either non-ICU or ICU group of COVID-19 cohort.

### Comparison of blood parameters between COVID-19 and influenza patients before treatment

For blood cell counts, a substantial or significant portion of patients in both cohorts demonstrated hematological abnormality, typified by leukocytosis, neutrophilia, lymphocytopenia, monocytosis, anemia and thrombocytopenia (Table 1). Compared to influenza cohort, COVID-19 cohort showed lessened severity in leukocytosis, neutrophilia and monocytosis, and increased severity in lymphocytopenia and anemia, whereas thrombocytopenia was found similar between both cohorts. Within the COVID-19 cohort, derangement in blood cell counts was generally associated with patient severity (except monocytosis) and mortality (except monocytosis and anemia) (Table 2). Using hematological data acquired upon admission, ICU patients showed worsened prognosis compared to non-ICU patients and likewise for non-survivors compared to survivors of ICU patients.

Notably, anemia was mirrored by low hemoglobin and low hematocrit, prevailing in patients of both cohorts (~ 60% or more), and thrombocytopenia was concurrent with heightened mean platelet volume (MPV), suggesting a dearth of platelets in the bloodstream and an overdriven production of nascent platelets by megakaryocytes in the bone marrow. Within COVID-19 cohort, a poor prognosis based on anemia was closely related to the severity (but not mortality), while thrombocytopenia turned out to be a risk factor for both COVID-19 severity and mortality (Table 2). Furthermore, influenza patients showed comparable degrees of anemia or thrombocytopenia to non-ICU COVID-19 patients (Additional file 1: Table S1).

Looking into coagulation factors, COVID-19 cohort demonstrated a longer PT and aPTT, higher INR, shorter thrombin time and lower fibrinogen, compared to those in influenza cohort (*p* < 0.05). However, heightened D-dimer levels were found similar between both cohorts (Table 1). Elevated D-dimer level has been reported as a prognostic indicator for COVID-19 death [11]. Our results agreed that heightened D-dimer levels had unfavored prognosis for both severity and mortality of COVID-19 infection (Table 2), and also indicated that viral infection in influenza patients induced abnormally high levels of D-dimer in analog to COVID-19. In addition, most coagulation factors showed prognostic values for COVID-19 severity but not mortality.

### Coagulation disorders and bleeding complications among COVID-19 and influenza patients after treatment

**Fig. 1:**
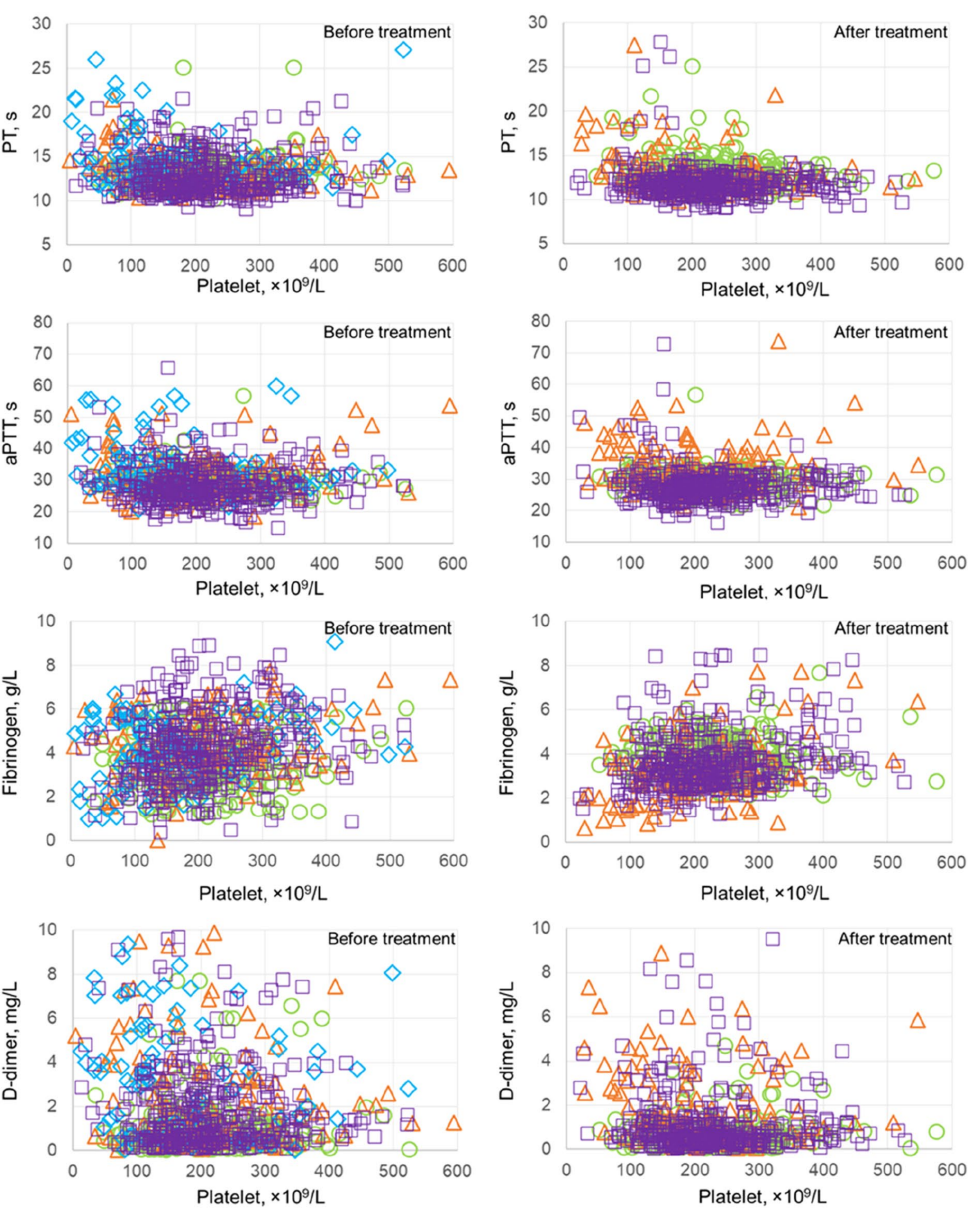
PT, aPTT, fibrinogen concentration, or D-dimer level was individually plotted versus the platelet count in each patient of influenza cohort (purple square) and COVID-19 cohort (green circle for the non-ICU group, orange triangle for the ICU survivors, and blue diamond for the ICU non-survivors), before (left) and after treatment (right). Data in the ICU non-survivors were not shown after treatment

For influenza patients, oxygen therapy was implemented with antiviral medications using ribavirin or oseltamivir, and none of them needed critical care, although 9 patients were unfortunately deceased in the non-ICU ward due to complications where influenza was not identified as a primary cause. After treatment, all hematological abnormalities (except anemia and prolonged PT/INR) were significantly mitigated (Table 3), showing a good treatability and recovery from seasonal influenza infection (Fig. 1).

**Table 3:** Blood parameters and coagulation factors of influenza patients are compared before and after treatment

Influenza	Normal range	Before treatment (n = 400)	After treatment (n = 400)	*p* value
*Blood cell counts*				
White blood cells, × 10^9^/L	3.5–9.5	7.3 (5.3–9.9)	6.5 (5.1–8.4)	< 0.0001
Neutrophils, × 10^9^/L	1.8–6.3	5.4 (3.6–7.7)	4.8 (3.4–6.6)	< 0.0001
Lymphocytes, × 10^9^/L	1.1–3.2	1.1 (0.8–1.5)	1.3 (0.9–1.8)	0.014
Monocytes, × 10^9^/L	0.1–0.6	0.5 (0.4–0.8)	0.5 (0.4–0.7)	0.002
Red blood cells, × 10^12^/L	4.3–5.8	4.2 (3.7–4.6)	4.2 (3.7–4.6)	0.212
Hemoglobin, g/L	130–175	127.0 (112.0–137.8)	126.0 (109.0–139.0)	0.988
Hematocrit, %	40–50	37.9 (33.9–41.7)	37.7 (32.8–41.0)	0.257
Platelet, × 10^9^/L	125–350	203.5 (152.3–267.0)	216.0 (166.0–275.5)	< 0.0001
Mean platelet volume, fL	7.4–12.5	10.7 (9.8–11.6)	10.6 (10.0–11.6)	0.405
*Coagulation panel*				
Prothrombin time, s	9–13	12.2 (11.4–14.1)	11.6 (10.9–12.4)	0.560
INR	0.8–1.2	1.0 (1.0–1.2)	1.1 (1.0–1.2)	0.645
aPTT, s	23.3–32.5	27.4 (24.7–31.3)	27.3 (24.9–29.7)	0.027
Thrombin time, s	14–21	17.7 (16.7–19.5)	17.5 (16.4–19.0)	< 0.0001
Fibrinogen, g/L	2–4	4.2 (3.2–5.3)	3.5 (2.8–4.1)	< 0.0001
D-dimer, mg/L	< 0.55	1.1 (0.5–2.5)	0.6 (0.4–1.2)	< 0.0001

For non-ICU COVID-19 patients, they were treated with broad-spectrum antibiotics including sulperazone and linezolid, and antiviral drugs including oseltamivir, arbidol and ribavirin. For COVID-19 ICU patients, they were receiving mechanical ventilation for respiratory support, while antibiotic, antiviral or/and antifungal therapeutics were also applied per necessity. Simultaneously, owing to the immobility of ICU patients, they were administered with daily LMWH unless bleeding risks were determined. As a result, many blood parameters in non-ICU COVID-19 patients were significantly ameliorated, especially fibrinogen and D-dimer levels, although discharged patients still showed common anemia (Table 4). In contrast, for ICU survivors, most hematological abnormalities did not exhibit substantial improvement upon hospital discharge, including anemia, thrombocytopenia, and most coagulopathy, although fibrinogen levels significantly dropped (Fig. 1).

**Table 4:** Blood parameters and coagulation factors of COVID-19 patients are compared before and after treatment in the non-ICU group and in the survivors of ICU group, respectively

COVID-19	Normal range	Non-ICU (n = 252)^*p1*^			ICU survivor (n = 119)^*p2*^		
		Before treatment	After treatment	*p1*	Before treatment	After treatment	*p2*
*Blood cell counts*							
White blood cells, × 10^9^/L	3.5–9.5	6.1 (4.7–7.5)	6.3 (5.3–7.4)	0.019	7.0 (4.9–10.1)	7.0 (5.0–9.3)	0.706
Neutrophils, × 10^9^/L	1.8–6.3	4.3 (2.9–5.9)	5.0 (3.8–6.1)	0.001	5.1 (3.4–7.8)	4.5 (2.9–6.8)	0.031
Lymphocytes, × 10^9^/L	1.1–3.2	1.1 (0.8–1.6)	1.5 (1.1–1.8)	< 0.0001	0.8 (0.5–1.3)	1.4 (1.0–1.9)	< 0.0001
Monocytes, × 10^9^/L	0.1–0.6	0.5 (0.3–0.6)	0.5 (0.4–0.6)	0.069	0.4 (0.3–0.6)	0.5 (0.4–0.7)	0.008
Red blood cells, × 10^12^/L	4.3–5.8	4.3 (3.9–4.6)	4.3 (4.0–4.7)	0.136	3.5 (2.9–4.1)	3.5 (2.8–4.2)	0.569
Hemoglobin, g/L	130–175	128.0 (115.3–140.0)	136.0 (121.3–146.0)	< 0.0001	108.0 (88.0–125.0)	97.0 (81.0–121.0)	0.010
Hematocrit, %	40–50	37.8 (34.6–40.9)	39.4 (36.1–42.5)	< 0.0001	32.5 (26.5–37.2)	31.0 (25.8–37.4)	0.365
Platelet, × 10^9^/L	125–350	201.0 (152.0–260.0)	239.5 (190.0–283.0)	< 0.0001	197.0 (140.0–276.0)	189.0 (135.0–274.0)	0.567
Mean platelet volume, fL	7.4–12.5	10.5 (9.7–11.2)	10.6 (10.2–11.4)	0.058	10.7 (10.2–11.5)	10.6 (10.0–11.7)	0.074
*Coagulation panel*							
Prothrombin time, s	9–13	13.4 (12.6–14.0)	12.7 (11.8–13.7)	< 0.0001	12.7 (11.7–13.9)	12.5 (11.6–14.0)	0.613
INR	0.8–1.2	1.1 (1.0–1.1)	1.1 (1.0–1.2)	0.300	1.1 (1.0–1.2)	1.1 (1.0–1.2)	0.007
aPTT, s	23.3–32.5	30.1 (28.1–31.5)	29.9 (27.8–31.5)	0.621	30.7 (28.0–35.3)	32.4 (28.5–38.4)	0.079
Thrombin time, s	14–21	15.9 (15.0–17.0)	15.6 (14.4–16.6)	0.082	17.1 (16.3–18.2)	17.5 (16.3–19.4)	0.343
Fibrinogen, g/L	2–4	3.5 (2.6–4.3)	3.7 (3.2–4.3)	< 0.001	4.2 (3.4–5.4)	3.0 (2.4–3.8)	< 0.001
D-dimer, mg/L	< 0.55	0.6 (0.2–1.2)	0.5 (0.2–0.8)	< 0.0001	2.2 (0.8–5.2)	1.6 (0.8–3.8)	0.160

Bleeding events were also observed in both cohorts during hospitalization, and major bleeding was identified to include bronchoalveolar, gastrointestinal, mucocutaneous and intracerebral hemorrhage (from high to low incidence) (Table 5). Hemostatic drugs (tranexamic acid or/and hemocoagulase) were applied to treat patients who bled, and plasma, cryoprecipitate, or platelet transfusion was applied once necessary. For COVID-19 cohort, bleeding was significantly more frequent in the ICU group than in the non-ICU group, but the bleeding occurrence was similar between survivors and non-survivors within ICU group (Additional file 1: Table S3). Consequently, tranexamic acid and component transfusion were applied more often in the ICU group when compared to the non-ICU group. Overall, influenza cohort exhibited comparable bleeding incidences at major hemorrhagic sites to COVID-19 cohort, except for bronchoalveolar bleeding where COVID-19 patients had much higher frequency, and administration of hemostatic drugs or component transfusion showed similar frequency between two cohorts. However, compared to either the influenza cohort or the non-ICU COVID-19 group, the ICU group in COVID-19 cohort exhibited significantly higher bleeding occurrence, more usage of hemostatic drugs and more needs for component transfusions.

**Table 5:** Comparison of bleeding events, usage of hemostatic drugs or component blood transfusion between the COVID-19 cohort and the influenza cohort

	Total (n = 870)	COVID-19 (n = 461)	Influenza (n = 409)	*p* value
*Bleeding events*				
Bronchus	25 (2.9%)	21 (4.6%)	4 (1.0%)	0.002
Gastrointestinal tract	24 (2.8%)	16 (3.5%)	8 (2.0%)	0.173
Mucocutaneous membrane	15 (1.7%)	11 (2.4%)	4 (1.0%)	0.125
Intracerebral hemorrhage	8 (0.9%)	7 (1.5%)	1 (0.2%)	0.073
*Hemostatic drugs*				
Tranexamic acid	37 (4.3%)	25 (5.4%)	12 (2.9%)	0.069
Hemocoagulase	16 (1.8%)	11 (2.4%)	5 (1.2%)	0.219
*Transfusion*	21 (2.4%)	15 (3.3%)	6 (1.5%)	0.087

## Discussion

Both SARS-CoV-2 and influenza viruses are enveloped particles that enclose viral nucleoproteins and single-stranded RNAs [19]. However, two viruses differ in many ways, such as their viral architectures, invasion patterns, and pathogenic mechanisms. With strike (S) proteins poking out on surface, SARS-CoV-2 is embedded with non-segmented positive-sense genomic RNAs (gRNAs), binding to ACE2 receptors on the host for cell entry that is facilitated by serine protease TMPRSS2 for S protein priming [5]. The internalized virus releases its gRNAs into the cytoplasm, borrowing cellular ribosomes to produce polyproteins that further undertake proteolysis for activation, responsible for the next viral RNA modification, replication, translation into nascent viral components (i.e., structural proteins and gRNAs) and assembly into virions [21]. In contrast, influenza viruses employ two surface proteins for cell entry, haemagglutinin (HA) and neuraminidase (NA), by attaching to sialic acids (SAs) as host cell receptor [22]. After endocytosis, the encapsulated viral proteins and eight negative-sense RNA segments are unleashed into cytoplasm, where RNAs were relocated into nucleus for conversion into positive-stranded mRNAs, being further transported into cytosols for viral protein synthesis and new virion assembly before budding [23].

SA owns a broad variety on different cell types throughout the human body, while ACE2 is actively expressed along the respiratory tract, including nasal epithelium, airway, and alveolar epithelium [24–26]. Additionally, human coronavirus S glycoproteins possess SA-binding sites [27], and SARS-CoV-2 owns a capacity to bind to SAs [28], but with a much weaker strength than its binding to ACE2 [29]. Those findings may point out another pathogenicity of COVID-19, although more evidences are needed. Concomitantly, pre-infection by influenza viruses can augment ACE2 expression, increasing further infectivity of SARS-CoV-2 and causing more severe pulmonary damage [30]. As the flu seasons overlap with COVID-19 pandemic, preventive measures and/or timely vaccinations against both respiratory viruses are imperative.

The clinical symptoms of COVID-19 include fever, cough, dyspnea, fatigue, diarrhea and vomiting, presenting an influenza-like illness [19]. In this study, hypertension, diabetes, cardiovascular diseases and bronchitis attributed to the leading comorbidities for both infections. The median age of influenza patients was statistically higher than that of COVID-19 patients. This is in consistency with our previous finding [19], but in controversy with some of others who reported younger ages of seasonal influenza patients, especially among children [31, 32]. The reason may lie in that young influenza patients with mild symptoms in China are reluctant to go to hospital, while suspected COVID-19 patients are obligatory to be tested and hospitalized if positive. Like high age, male gender is another risk factor for COVID-19 severity and mortality, although the exact mechanism requires further investigations and opposite reports exist [33]. In general, age and gender biases have been reported among many viral diseases, and this could be associated with at least two major aspects; one contains socioeconomic traits including social and cultural traditions, living styles (e.g., smoking history) and occupational experiences, etc., and the other dedicates to biological features, exemplified by differences in age- or sex-related hormones and immune responses [33, 34].

Compared to the influenza cohort, the COVID-19 cohort showed similar frequency of leukocytosis and anemia, less frequency of neutrophilia and monocytosis, but more frequency of lymphocytopenia and thrombocytopenia. This result showed some variation from our previous observation in a different cohort study, but stood in line with others [35]. The frequencies of irregular cell counts in the patients’ blood might fluctuate if small pools of data are selected. For COVID-19 patients, leukocytosis, neutrophilia, lymphocytopenia, and thrombocytopenia were associated with disease severity and mortality, being characteristic of SARS-CoV-2 infection [36, 37]. Simultaneously, the prevalence of anemia was found similar between COVID-19 and influenza cohorts. After treatment, most derangements in blood cell counts were significantly alleviated in influenza and COVID-19 non-ICU patients (except anemia), but at time of hospital discharge many hematological parameters (e.g., anemia and thrombocytopenia) in COVID-19 ICU survivors did not demonstrate noticeable improvement.

Being one of common manifestations, anemia has been recognized as a prognostic indicator of COVID-19 severity but not mortality, agreeing to our finding here [38, 39]. The underlying machinery could be complexed as direct and indirect hemolysis might be causative following viral infection. Directly, SARS-CoV-2 might interplay with CD147 receptor on RBCs as another cell entry route [40], leading to rupture of erythrocytes and loss of Hb. Indirectly, systemic inflammatory burden in COVID-19 patients after lung infection may result in further prothrombotic state, including complement activation, platelet hyperreactivity, and hypoxia in major organs [41]. Consequently, erythropoiesis can be suppressed in the bone morrow, and the circulating erythrocytes can be shattered, each causing anemia. In fact, anemia is not infrequent in viral or other microbial infections, such as community-acquired pneumonia and hepatitis viruses [42, 43]. However, in our study influenza-related anemia showed a less severity than COVID-19 one, supported by others [44]. Given the abundancy of SA on the surface of RBCs and its specificity for influenza virus binding, its direct hit on the erythrocytes can be impactful. Cytokine levels in influenza patients were reported no less than those in COVID-19 patients [45]. Therefore, both SARS-CoV-2 and influenza viruses could cause anemia in a comparative pattern but to a different extent.

Thrombocytopenia has been established with a prognostic value of COVID-19 severity and mortality [11, 46]. Recent study has revealed that lung is a primary location of terminal platelet biogenesis and a major reservoir for hematopoietic progenitors [47]. Migrating from extrapulmonary sites (e.g., bone marrow), the megakaryocytes and hematopoietic progenitors reside in the lung, generating ~ 50% of all platelets there before their release into circulation, and in case of thrombocytopenia, those progenitors displace outside the lung to re-supply platelets in the blood [47]. Rich ACE2 and TMPRSS2 expressions have been confirmed in platelets or platelet-producing megakaryocytes, suggestive of a direct viral hit [48]. Thus, the lung infection by SARS-CoV-2 can trigger double whammy on platelets, causing increased destruction and decreased production. This viremic effect undermined megakaryocytes and instigated platelet activation via mitogen-activated protein kinase (MAPK) pathway [48, 49]. Moreover, in a substantial portion of COVID-19 patients, thrombocytopenia was found together with prolonged PT/INR and aPTT, and elevated levels of fibrinogen and D-dimer, but change in thrombin time was marginal. Our results indicated a unique profile of coagulopathy, revealing a hypercoagulative and hypofibrinolytic state in the COVID-19 patients [46, 50, 51]. Consequently, platelets in COVID-19 patients tend to form aggregates with immune cells, including T cells, monocytes, and neutrophils, showing platelet hyperreactivity that contributes to immunothrombosis [49, 52, 53]. Autopsy findings confirmed the platelet–fibrin thrombi in the microvasculature of major organs, including lung, heart, kidney and liver, with or without detectable virions [54]. Hence, immunity-mediated thrombotic events in COVID-19, predominantly in lungs and sporadically in other organs, are closely associated with disease severity and mortality [55].

Similarly, influenza viruses have been previously reported for their detrimental interaction with platelets, manifested as thrombocytopenia, elongated aPTT and elevated D-dimer [56]. Influenza infection stimulates cytokine responses in patients, represented as heightened levels of tumor necrosis factor (TNF)-a and interleukin(IL)-6, activating platelets and forming aggregates with immune cells (e.g., neutrophils and monocytes) [57, 58]. Accrediting to viral pathogenicity, thrombocytopenia and platelet activation depend on influenza virus subtype and SA receptor in the platelets, which induce coagulation disorders to various degrees and lead to thrombotic vascular occlusion [59, 60]. In severe pneumonia induced by either seasonal or pandemic influenza viruses, disseminated intravascular coagulation (DIC) has been found in patients with multiple organ dysfunction syndrome (MODS) and acute respiratory distress syndrome (ARDS), whose anticoagulant (i.e., heparin) treatment lessened severity or improved survival [18].

In contrast, COVID-19 patients demonstrated more intricate thrombotic complications, encompassing low-grade DIC, localized thrombotic microangiopathy (TMA), vein thromboembolism (e.g., deep vein thrombosis) and arterial thrombosis [61]. Notably, COVID-19 patients with ARDS displayed much higher platelet hyperreactivity than non-COVID-19 patients with ARDS of equal severity [62]. Compared to the influenza cases, inflammation- and angiogenesis-linked genes that were exclusively altered in COVID-19 patients were found ~ 40 times and > 2 times higher, respectively, suggestive of more profound perivascular inflammation and endothelial injury [63]. As a result, the burden of platelet-rich microthrombi in alveolar capillary of COVID-19 patients was 9 times heavier, and the presence of intussusceptive angiogenesis was ~ 3 times more [63]. A clinically important and rare coagulopathy, antiphospholipid autoantibody syndrome was found in COVID-19 patients [64], possibly due to the impaired platelets or/and RBCs turned negatively charged phosphatidylserine of the inner cellular membrane inside out [65], producing antibodies against phospholipids in autoimmune responses. This further triggered platelet activation and thrombin formation, complicating COVID-19-related thrombosis. Recently, a comprehensive comparison on risks of clinical characteristics and mortality patients indicated much higher risks of thrombotic events and death in COVID-19 patients than seasonal influenza patients [66], whereas increased risks of thrombosis were highly associated with elevated mortality in COVID-19 patients [67].

Antiviral treatment in seasonal influenza patients with no LMWH administration significantly alleviated thrombocytopenia and most of coagulopathy (except PT), and improved their clinical outcomes. Similarly, after treatment, coagulation dysfunctions including thrombocytopenia, fibrinogen shutdown, and D-dimer elevation in COVID-19 non-ICU patients was significantly improved at the time of hospital discharge; however, despite of LMWH usage in the COVID-19 ICU patients, their coagulopathy remained severe (except mitigated fibrinogen levels), implying persistent thrombosis risks in critically ill patients after hospital discharge. This result was corroborated by others in that 1–3 months after hospital discharge a portion of COVID-19 patients still showed symptoms or were even diagnosed with thrombotic complications, especially for those survived the critical care [68–70]. However, the rate of COVID-19 postdischarge thrombosis is low and non-specific as compared to other acute illness [69].

Finally, yet importantly, our results indicated a higher bleeding incidence during hospitalization in the severe COVID-19 patients who received anticoagulation medication, than either non-severe COVID-19 patients or the influenza patients who did not received anticoagulation. This is supported by other reports where similar frequencies of thrombosis and bleeding in COVID-19 patients were observed, comparable to the situations in other critical illness of similar severity, and this in-hospital bleeding might be associated with the intensified anticoagulant treatment [71–73]. Moreover, the hemorrhagic rate was also found close to that of thrombosis after months following hospital discharge [68, 70].

The study has some limitations. First, our conclusion might be limited by patient number and case availability. Although 461 patients included in this study might be representative of COVID-19 cases in Hubei province during the early outbreak in China, the disease development and the patient outcome could be changing as management and treatment of COVID-19 are quickly evolving worldwide. For 409 seasonal influenza patients, given the fact that the ratio of severe patients in general population is low, especially in a non-epidemic period, only mild to moderate patients who did not require critical care are available and included in this study. Second, due to the emergency of COVID-19 as a major infectious disease, many laboratory tests were unavailable on spot, such as ultrasonography to diagnose possible pulmonary embolism in critically ill patients, and some critical parameters in monitoring hematological changes of patients over time were absent, such as coagulation and cytokine profiles. This would otherwise elucidate more about the coagulopathy of this deadly viral disease.

Put together, our results point out that the platelet injury by SARS-CoV-2 infection with consequence of coagulopathy and thrombi formation in severe COVID-19 patients may take responsibility for the disease fatality and sustain a longstanding prothrombotic state in COVID-19 survivors. Compared to influenza cases, platelet hyperreactivity in COVID-19 is more evident, associated with worse hyper-inflammatory response and more refractory coagulopathy. Early antibiotic and antiviral interventions in COVID-19 patients could efficiently attenuate the coagulation disorders, and thromboprophylaxis together with immunomodulatory medications (such as inflammation reducers) in severe patients may help dampen the platelet reactivity, so lessening the severity and lowering the mortality. However, as bleeding complications occur in hospitalized COVID-19 patients and may continue after hospital discharge, extended thromboprophylaxis for COVID-19 survivors should be carefully evaluated.

## Supplementary Information

**Additional file 1: Table S1.** Baseline characteristics, blood parameters and coagulation factors in the non-ICU and the ICU groups of COVID-19 cohorts, compared to those in the influenza cohort, respectively. **Table S2.** Comparison of blood parameters within the COVID-19 cohort between the non-ICU group and the ICU survivors before and after treatment, respectively. **Table S3.** Comparison of bleeding events, usage of hemostatic drugs or component transfusion within the COVID-19 cohort between the non-ICU and the ICU groups, between survivors and non-survivors in the ICU groups, respectively. In addition, the non-ICU or the ICU groups of COVID-19 cohort was also compared to the influenza cohort, respectively. **Figure S1.** Frequency of each blood cell count and coagulation character was plotted between (A) COVID-19 cohort versus influenza cohort; (B) ICU group versus non-ICU group within the COVID-19 cohort; (C) non-survivors versus survivors within the COVID-19 ICU group. Diagonal line (dotted) indicated a hypothetically equal frequency between two groups.

## Data Availability

Data available on request due to privacy/ethical restrictions.
