# Supplementary material for: Platelet-driven coagulopathy in COVID-19 patients: in comparison to seasonal influenza cases
Source: Exp Hematol Oncol. 2021 May 31;10:34. doi: 10.1186/s40164-021-00228-z (PMC8165133; doi:10.1186/s40164-021-00228-z)

Additional Information

for

**Platelet-driven coagulopathy in COVID-19 patients:**

**in comparison to seasonal influenza cases**

Jianguo Zhang^1,2, †^, Xing Huang^3, †^, Daoyin Ding^4, †^, Zhimin Tao^1, *^

^1^Jiangsu Province Key Laboratory of Medical Science and Laboratory Medicine, School of Medicine, Jiangsu University, Zhenjiang, Jiangsu 212013, China

^2^Department of Critical Care Medicine, The Affiliated Hospital, Jiangsu University, Zhenjiang, Jiangsu 212001, China

^3^Center for Evidence-based and Translational Medicine, Zhongnan Hospital of Wuhan University, Wuhan 430071, China

^4^Department of Critical Care Medicine, The First People’s Hospital of Jiangxia District, Wuhan, Hubei 430200, China

^†^Jianguo Zhang, Xing Huang, and Daoyin Ding contributed equally to this work.

^*^ Correspondences should be addressed to:

Zhimin Tao: [jsutao@ujs.edu.cn](mailto:jsutao@ujs.edu.cn)

**Table S1.** Baseline characteristics, blood parameters and coagulation factors in the non-ICU and the ICU groups of COVID-19 cohorts, compared to those in the influenza cohort, respectively.


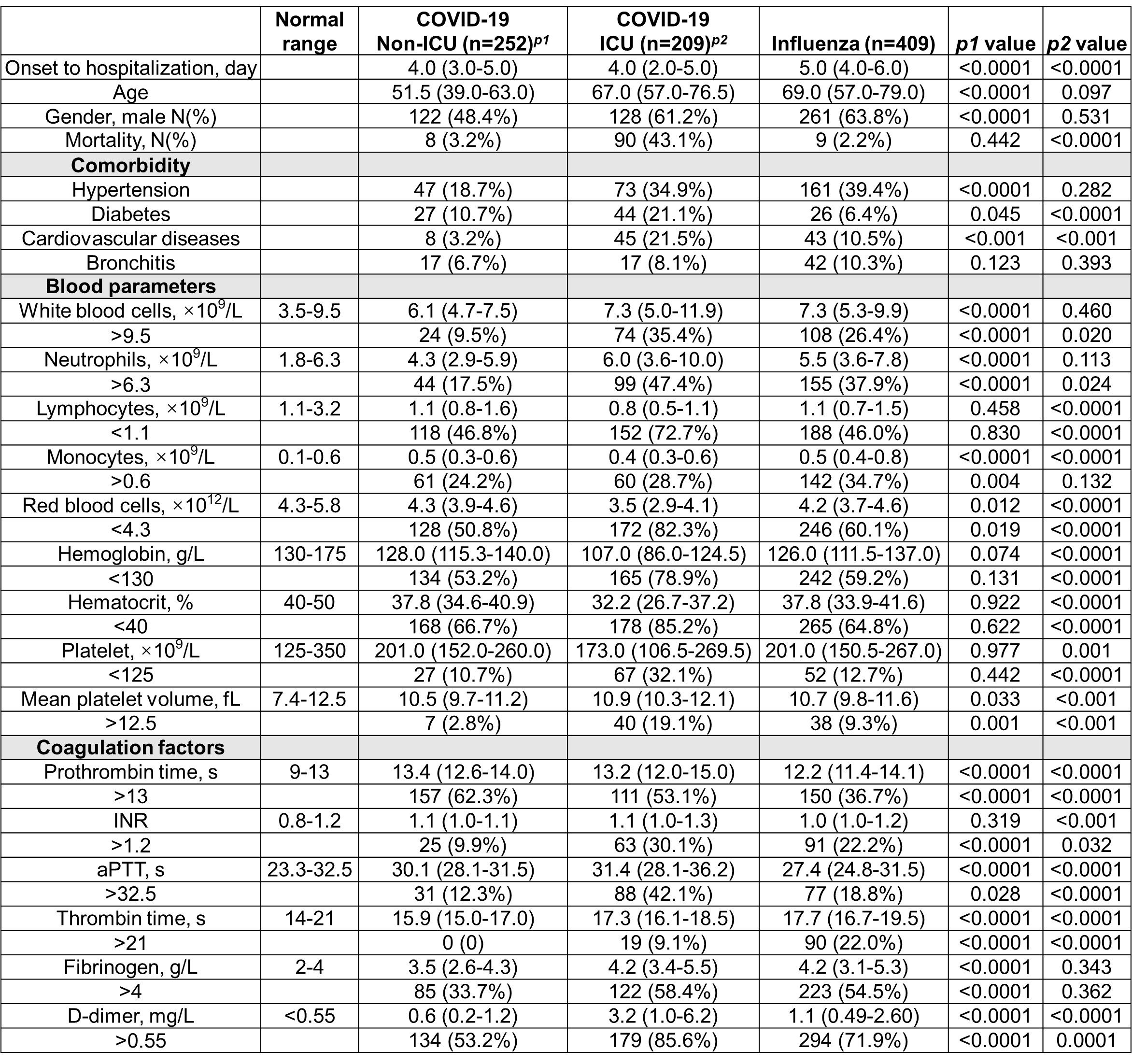


**Table S2.** Comparison of blood parameters within the COVID-19 cohort between the non-ICU group and the ICU survivors before and after treatment, respectively.

**
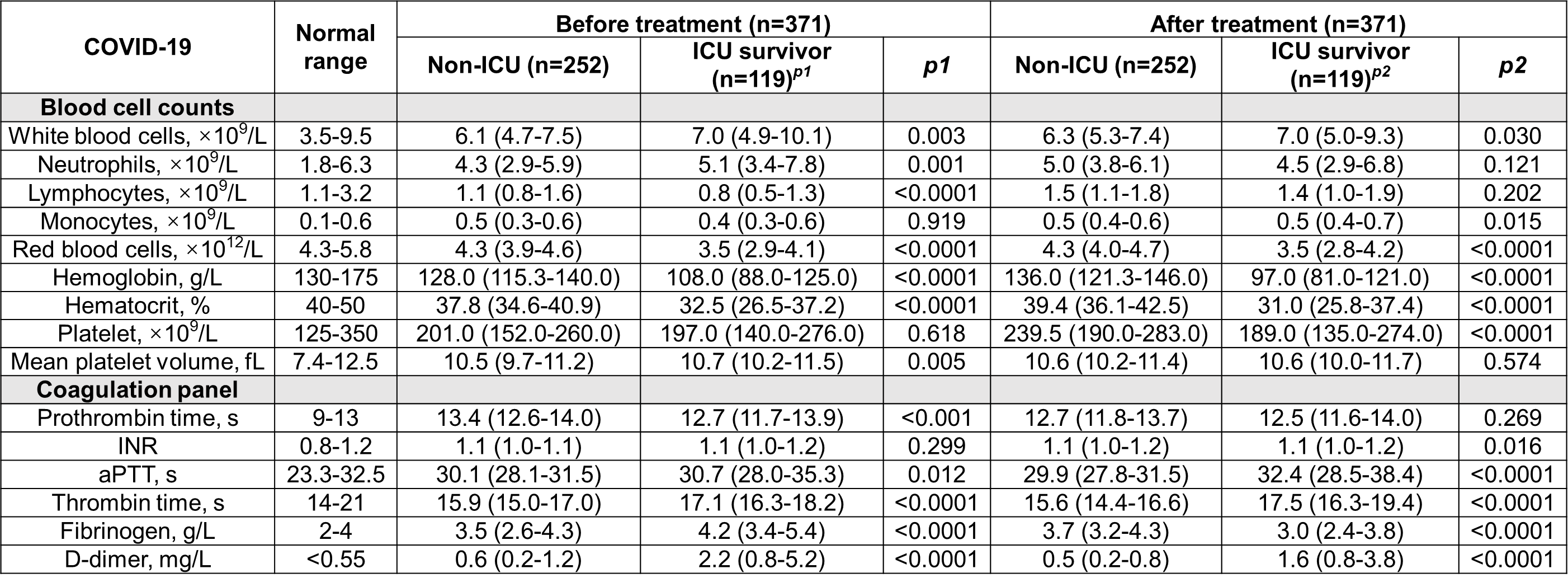
**

**Table S3.** Comparison of bleeding events, usage of hemostatic drugs or component transfusion within the COVID-19 cohort between the non-ICU and the ICU groups, between survivors and non-survivors in the ICU groups, respectively. In addition, the non-ICU or the ICU groups of COVID-19 cohort was also compared to the influenza cohort, respectively.

**
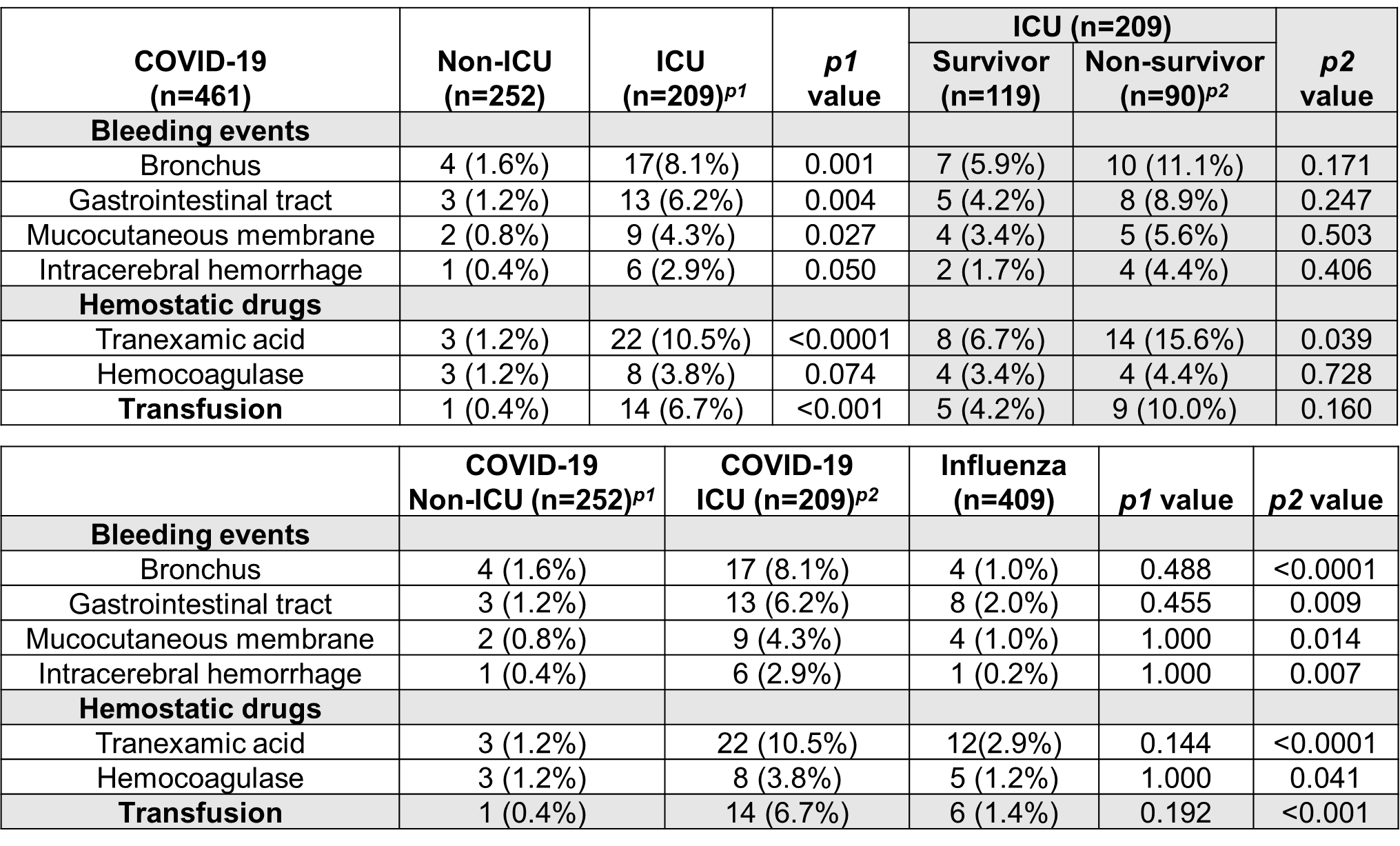
**

**
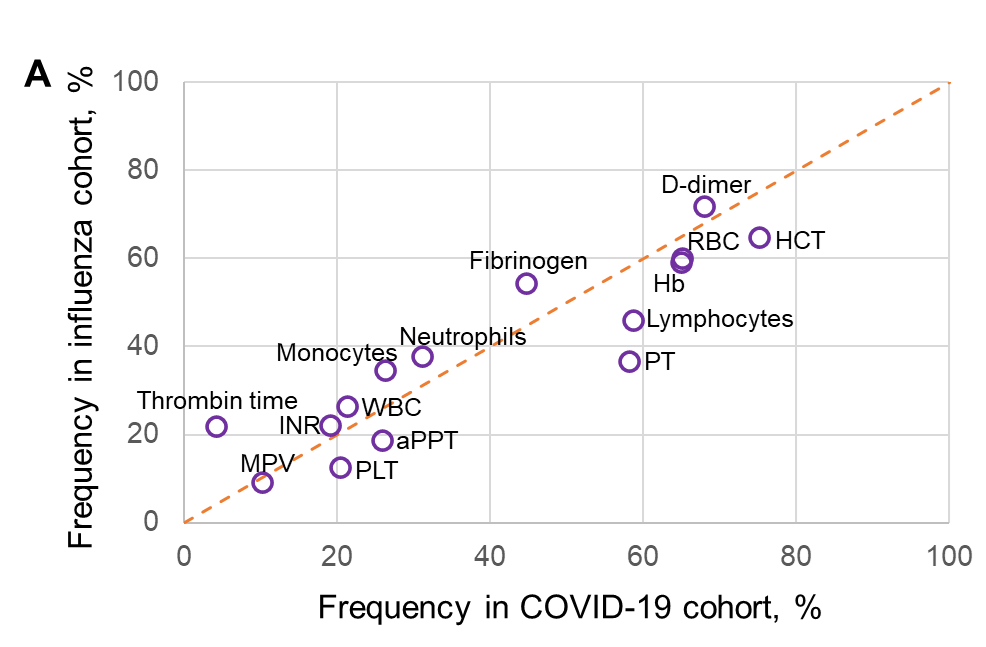
Figure S1.** Frequency of each blood cell count and coagulation character was plotted between (A) COVID-19 cohort versus influenza cohort; (B) ICU group versus non-ICU group within the COVID-19 cohort; (C) non-survivors versus survivors within the COVID-19 ICU group. Diagonal line (dotted) indicated a hypothetically equal frequency between two groups.


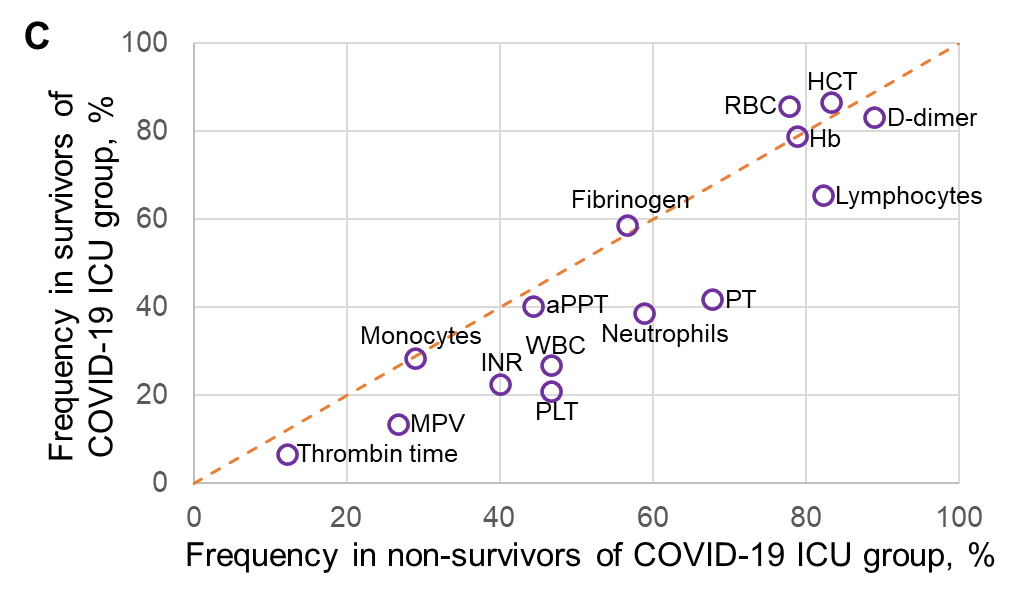

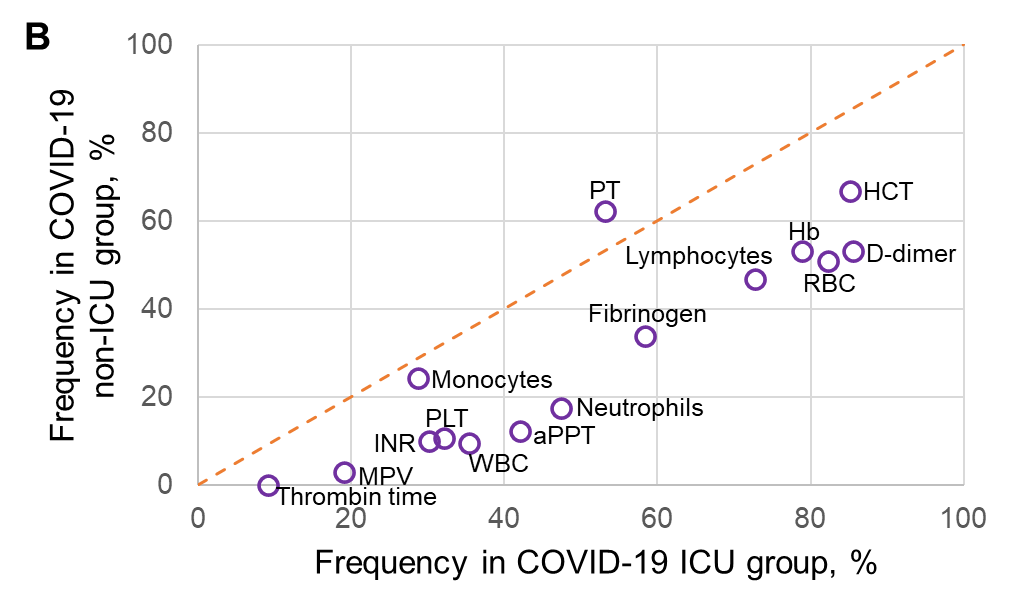

Supplement: Supplementary file 1 — Additional file 1: Table S1. Baseline characteristics, blood parameters and coagulation factors in the non-ICU and the ICU groups of COVID-19 cohorts, compared to those in the influenza cohort, respectively. Table S2. Comparison of blood parameters within the COVID-19 cohort between the non-ICU group and the ICU survivors before and after treatment, respectively. Table S3. Comparison of bleeding events, usage of hemostatic drugs or component transfusion within the COVID-19 cohort between the non-ICU and the ICU groups, between survivors and non-survivors in the ICU groups, respectively. In addition, the non-ICU or the ICU groups of COVID-19 cohort was also compared to the influenza cohort, respectively. Figure S1. Frequency of each blood cell count and coagulation character was plotted between (A) COVID-19 cohort versus influenza cohort; (B) ICU group versus non-ICU group within the COVID-19 cohort; (C) non-survivors versus survivors within the COVID-19 ICU group. Diagonal line (dotted) indicated a hypothetically equal frequency between two groups. [file 40164_2021_228_MOESM1_ESM.docx]
